# Resection with intraoperative radiotherapy vs. adjuvant radiotherapy in the treatment of eloquent brain metastases: an analysis of feasibility and safety

**DOI:** 10.1007/s10143-025-03523-z

**Published:** 2025-04-24

**Authors:** Philipp Krauss, Micol Colosimo, Christina Wolfert, Bastian Stemmer, Bjoern Sommer, Dorothee Mielke, Georg Stueben, Klaus Henning Kahl, Ehab Shiban

**Affiliations:** 1 Department of Neurosurgery, University Hospital Augsburg, Stenglinstrasse 2, 86156 Augsburg, Germany; 2 Department of Radiooncology, University Hospital Augsburg, Stenglinstrasse 2, 86156 Augsburg, Germany; 3 BZKF, Bayerisches Zentrum für Krebsforschung, Augsburg, Germany; 4 Department of Neurosurgery, Medical University Lausitz – Carl Thiem, Thiemstrasse 111, 03048 Cottbus, Germany

**Keywords:** Intraoperative radiotherapy, Eloquent brain tumor, Neurooncology, Brain metastasis, Neurosurgery

## Abstract

**Introduction:**

The treatment of motor eloquent brain metastases (BM) harbors an elevated risk of neurological deficits due to possible damage to motor-cortex and tracts. Preserving a good functional and neurological status is crucial to enable comprehensive oncologic treatment. Growing evidence promotes intraoperative radiotherapy (IORT) with low voltage x-rays as alternative to adjuvant external beam radiotherapy (EBRT). Aim of this study is to investigate the safety and feasibility of surgery with IORT in motor eloquent regions compared to adjuvant radiotherapy (RT).

**Methods:**

We performed a retrospective chart review analysis of patients undergoing surgery for motor eloquent BMs at our institution with either IORT or adjuvant RT. All patients were resected under intraoperative neuromonitoring (IONM). We compared patient characteristics, the rate of neurological deficits along with IONM parameters, functional status (KPS) and adverse events (AE) in both groups.

**Results:**

33 patients were analyzed from which 25 underwent IORT and 8 adjuvant EBRT in motor eloquent BMs. New motor deficits occurred in 7/33 patients without significant difference between both groups after 30 days (IORT 4/25 vs. adj. RT 3/8; Chi [2]-test: *p* = 0.19). The KPS after surgery did not differ significantly between both groups (IORT: 90% [72.5–90] vs. adj. RT: 80% [70–90]; Mann-Whitney-U-test: *p* = 0.31). No patient experienced local tumor recurrence or radio necrosis. 9/33 patients experienced postoperative AEs until the 30 day follow up without significantly different rates between both groups (IORT 5/25 vs. adj. RT 4/8; Chi [2]-test: *p* = 0.09).

**Conclusion:**

50 kV photon IORT is a safe treatment option for motor eloquent BMs and does not seem to provoke in symptomatic brain irritation.

## Introduction

Treatment of motor eloquent brain lesions can be challenging due to possibly deleterious neurological deficits [1]. In the treatment of brain metastases (BM), various considerations have to be taken into account if planning an individual treatment strategy [2]. In case of resection for motor eloquent BMs, neurological deficits can highly impact the patients quality of life and delay the further comprehensive treatment. Regular treatment algorithms include resection of symptomatic lesions, followed by radiotherapy (adj. RT) of the resection cavity and systemic treatment [2, 3]. Intraoperative radiotherapy (IORT) has developed as promising alternative to adj. RT in the treatment of BMs [4, 5]. This technique has been shown to be safely feasible and reduce the time to systemic treatment in metastatic disease [6, 7, 8, 9]. New neurological deficits can have severe consequences and delay the further oncologic treatment. These deficits in motor eloquent regions of the brain can result from mechanical destruction of motor-cortex and tracts, ischemia, venous congestion, epileptic seizures or symptomatic edema. However, there is uncertainty if IORT in highly eloquent areas of the brain can induce symptoms due to irritation of neural structures. The aim of this study is to investigate the safety of IORT in the resection of motor eloquent BM according to neurological deficits and adverse events.

## Methods

### Study design

We performed a retrospective analysis of patient-specific clinical records at a single tertiary neurosurgical center. The primary outcome was defined as new motor deficit 30 days after surgery (with IORT or without IORT) in order to evaluate the risk of adding IORT to surgery for motor eloquent lesions. Further parameters were age, Karnofsky Performance Scale (KPS) before surgery, after surgery and after radiotherapy, Recursive partitioning analysis (RPA), length of surgery (LOS), motor deficit before and directly after surgery, tumor- size and entity, radiation dose and fractions, time to RT, radio necrosis until the last follow up, steroid dose at discharge and adverse events according to the Clavien Dindo Grading system (CDG) until 30 days after surgery [10, 11, 12]. Furthermore, decline of > 50% signal intensity from intraoperative electrophysiological monitoring was analyzed in every case.

### Patient selection

All adult patients who underwent resection of motor eloquent BMs and received either IORT or adj. RT of the resection cavity between February 2021 and September 2023 were included. The indication for resection was discussed and consented for every single case in the local interdisciplinary neurooncologic board. Generally if the tumor diameter was < 2.5 cm, in case of a solitary brain lesion or in case of a symptomatic mass effect due to tumor or edema or if new histologic specimen were needed, a recommendation for resection rather than stereotactic radiosurgery was chosen. Motor eloquence was defined as tumor within or near the primary motor cortex or the corticospinal tract. Patients that did not have resection under IONM, were under the age of 18 or underwent stereotactic biopsy alone were excluded from the analysis.

As part of the institutional standard operating procedure from 2021 on, all patients with eloquent tumors should undergo resection with IONM and IORT should be offered to all patients with BMs. Patients that did not undergo surgery with IORT did either refuse IORT or technical circumstances made IORT impossible.

### Intraoperative radiotherapy

Indication for treatment was confirmed by the local multidisciplinary tumor board in all cases. IORT was offered routinely as an alternative to postoperative external-beam RT following an expert panel of the German Society for Radiation Oncology (DEGRO) guideline [Expert panel decision DEGRO, inquiry 123, 17.02.2017]. Patients were considered ineligible if (1) the distance between the border of the MRI contrast-enhancing lesion and the brainstem was < 5 mm, (2) there was a history of small-cell lung cancer or (3) the resection trajectory was estimated to not allow a safe introduction of the radiation applicator. All patients signed informed consent for resection and IORT. After tumor extirpation, the resection cavity was irradiated with 50-kV x-rays via an INTRABEAM system (ZEISS MEDITEC AG, Oberkochen, Germany). The device and procedure have been described previously [7, 13]. A suitable spherical applicator was installed according to the size of the resection cavity, providing direct contact of the cavity walls to the surface of the applicator. Radiation dose (20 Gy) was prescribed to the surface of the applicator corresponding to the target volume/dose concept of postoperative SRS cavity treatment (GTV = CTV = cavity).

### Adjuvant external beam radiotherapy

Adjuvant radiotherapy consisted of a linac based fractionated stereotactic EBRT with 5 fractions of 6 Gy or 5 fractions of 7 Gy (in case of suspected incomplete resection on postoperative MRI scan). The dose was prescribed to the resection cavity with a 3 mm margin. Immobilization was performed with a thermoplastic mask system. Planning CT was reconstructed in 1 mm slices. Adj. RT was administered in absence of wound healing issues, systemic infection and good functional status (KPS ≥ 70%) as inpatient or outpatient procedure according to the patients’ general status.

### Intraoperative neuromonitoring

All recordings are taken using a 16-channel Inomed ISIS system^®^. Due to the significant impact of inhaled halogenated anesthetics on IONM, a total intravenous anesthetic (TIVA) protocol is utilized to facilitate IONM. Neuromuscular blocking is avoided during the procedure. Muscle action potentials are measured via subdermal needle electrodes placed in a bipolar fashion (M. Abductor pollicis brevis and M. biceps brachii for the upper extremity, M. tibialis anterior and M. adductor hallucis longus for the lower extremity). Transcranial stimulation is performed with a train of 5 pulses at 300 Herz (Hz). Stimulation intensity is raised from 10 mA on until a robust muscle response is seen. Motor evoked potentials (MEP) are continuously recorded and analyzed according to latency and amplitude every 50 s referenced to a baseline after dural opening until dural closure, During IORT the recording is paused because all personal stays outside the operation room for the time of radiation. A decline in amplitude of > 50% is immediately reported to the surgeon, resection is halted and the surgical field is irrigated with saline solution. After stabilization of the potentials, the resection is continued.

### Statistics

Statistical analysis was performed using the software SPSS Statistics™ (version 25, IBM Corp, Armonk, New York, USA). Normal distribution was evaluated according to the central limit theorem. Data was analyzed with an unpaired Mann- Whitney U-test, dichotomous variables were analyzed by means of Chi [2]-test. Data in text and graphs are shown as mean and standard deviation (SD) for continuous data and as median and interquartile range for ordinal data. A *p* value ≤ 0.05 was considered significant and indicated by “*”, *p* values ≤ 0.01 were indicated by “**,” and values ≤ 0.001 by “***.”

### Ethics approval

The study protocol was approved by the local institutional ethics committee (LMU: 23–0845) in accordance to the Declaration of Helsinki. For this retrospective observational study, no individual informed consent was necessary according to the ethics committee’s guidelines and regulations. Clinical Trial Number: not applicable.

### Availability of materials and data

Data is available upon request.

## Results

### Patient population

In this study, a total of 33 patients was analyzed, with 25 of them undergoing surgery with IORT and 8 surgery with adjuvant RT. Baseline characteristics did not differ significantly among both groups (Table 1). Patients suffered from a variety of oncologic diseases (Table 2). The mean follow up at the local comprehensive cancer center was 275 ± 265 days (median 182 [73–480)] after radiotherapy. The estimated mean volume of metastases was 33.8cm^3^ ± 44.1cm^3^. The metastases were located cortical (IORT *n* = 12, adj. RT *n* = 6) in 18 cases and subcortical (IORT *n* = 13, adj. RT *n* = 2) in 15 cases. The majority of BMs was located in the precentral gyrus (*n* = 10, IORT *n* = 7, adj. RT *n* = 3) and the supplementary motor area (SMA) (*n* = 10, IORT *n* = 7, adj. RT *n* = 3) followed by parietal lobe (*n* = 6, all IORT), postcentral gyrus (*n* = 4, IORT *n* = 2, adj. RT *n* = 2) and temporal (*n* = 1) and cerebellar (*n* = 1) lesions. In all patients, gross total resection was achieved and confirmed by postoperative cranial MRI. Using post hoc power analysis, we can assume a power of 80% with an alpha error of 0.05 at the current sample size (= 33) for a medium effect size (cohen’s *w =* 0.49).

**Table 1:** Baseline characteristics: y = year, RPA = recursive partitioning analysis, BM = brain metastasis, cm = centimeter, adj. RT = adjuvant radiotherapy, IORT = intraoperative radiotherapy, L = left, R = right, KPS = Karnofsky Performance Score, OP = surgery; data is shown as (mean ± SD / median [interquartile range]), A *p* value ≤ 0.05 was considered significant

	all	IORT	Adj. RT	significance
Number	33	25	8	
Age (y)	67.5 ± 10.1	66.5 ± 9.8	70.7 ± 10.9	0.42
Sex (f/m)	19 / 14	13 / 12	6 / 2	0.42
Tumor size (cm)	3.4 ± 1.5	3.5 ± 1.6	3.2 ± 1.1	0.89
BM Localization (L/R)	15 / 18	9 / 16	6 / 2	
KPS pre. OP (%)	90 [80–90]	90 [80–90]	85 [65–90]	0.29
RPA	2 [1.5-2]	2 [1.5-2]	2 [1.25-2]	0.64

**Table 2:** Tumor entity per group: adj. RT = adjuvant radiotherapy; IORT = intraoperative radiotherapy; NSCLC = non-small cell lung carcinoma; RCC = renal cell carcinoma; CRC = colorectal cancer

Oncologic disease	all	IORT	Adj. RT
NSCLC	14	11	3
Breast cancer	6	4	2
RCC	3	3	0
CRC	2	2	0
Other*	8	5	3

* sarcoma *n* = 1, pancreas *n* = 1, ovary *n* = 1, melanoma *n* = 1, prostate *n* = 1, sinunasal *n* = 1, urothel *n* = 1, esophagus *n* = 1

### Neurological outcome

**Fig. 1:**
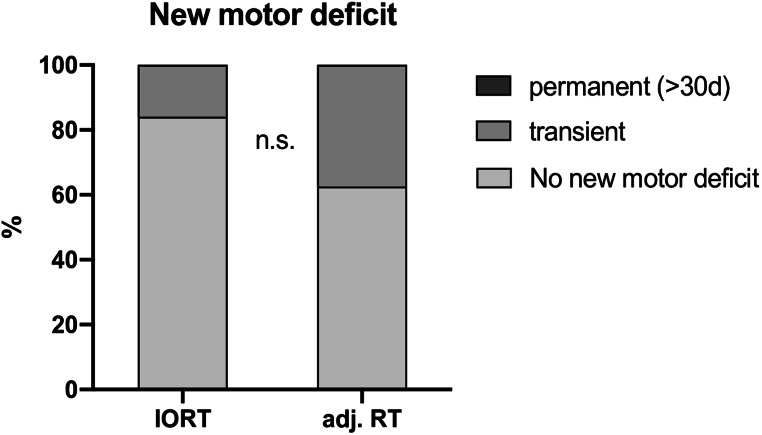
Rate of new postoperative motor deficits: dark grey = permanent, medium grey = transient < 30d, light grey = no new motor deficit; adj. RT = adjuvant radiotherapy, IORT = intraoperative radiotherapy; data is shown as (%), n.s. = not significant

Motor eloquent metastases were operated in all patients. A new corresponding motor deficit occurred in 7 out of 33 patients. Neither IORT nor adj. RT were attributed with higher rates of de novo postoperative motor deficits (IORT 4/25 vs. adj. RT 3/8; Chi [2]-test: *p* = 0.19) (Fig. 1). In 2 (1/25 IORT vs. 1/8 adj. RT) patients, no 30-day follow up motor evaluation was possible due to death during the postoperative course. In the patients that developed new postoperative motor deficits and received IORT, the BMs were located parietal subcortical (*n* = 1), the precentral gyrus (*n* = 1) and the postcentral gyrus (*n* = 2). In patients that received adj. RT with new motor deficits, the BMs were located in the precentral gyrus, the postcentral gyrus and the SMA (*n* = 1 each).

In both groups, no patient had a new motor deficit that persisted at the 30 day follow up after surgery. The appearance of a de novo motor deficit showed no significant association (Mann-Whitney-U-test) with age (*p* = 0.71), KPS before surgery (*p* = 0.31), RPA (*p* = 0.91), Tumor size (*p* = 0.48) or LOS (*p* = 0.62) (Table 3).

**Table 3:** Surgical and outcome parameters: LOS = length of surgery, min. = minutes, OP = surgery, mg = milligram, RT = radiotherapy, Gy = Gray, cm = centimeter, n = number, d = days, MEP 0 motor evoked potential, Y = yes, N = no, AE = adverse event, KPS = Karnofsky Performance Score, adj. RT = adjuvant radiotherapy, IORT = intraoperative radiotherapy; data is shown as (mean ± SD / median [interquartile range]), A *p* value ≤ 0.05 was considered significant

	all	IORT	Adj. RT	significance
Number	33	25	8	-
LOS (min.)	142 ± 52	150 ± 54	118 ± 39	0.19
Steroid dose post OP (mg)	9.6 ± 8.3	9.9 ± 8.4	8.5 ± 8.4	0.82
RT dose (Gy)	**22.8 ± 5.2**	**20 ± 0**	**31.6 ± 2.9**	**< 0.001**
RT applicator size (cm)	-	2.3 ± 0.7	-	-
RT fractions (n)	**1 [1–2]**	**1 [1–1]**	**5 [5-8.75]**	**< 0.001**
Time to RT start (d)	**8.9 ± 17.2**	**0 ± 0**	**37 ± 13**	**< 0.001**
Time to RT end (d)	**10.7 ± 20.2**	**0 ± 0**	**44 ± 13**	**< 0.001**
MEP decline (Y/N)	9 / 24	8 / 17	1 / 7	0.28
MEP decline reversible (Y/N)	8 / 1	7 / 1	1 / 0	0.37
New paresis post OP (Y/N)	7 / 26	4 / 21	3 / 5	0.19
New paresis post 30 d (Y/N)*	0 / 29	0 / 22	0 / 7	0.42
Patients with AE (Y/N)	9 / 20	5 / 20	4 / 4	0.09
KPS post OP (%)	85 [70–90]	90 [72.5–90]	80 [70–90]	0.31
KPS post RT (%)	90 [57.5–90]	90 [72.5–90]	90 [57.5–90]	0.64
Radionecrosis (Y/N)	0 / 29	0 / 22	0 / 7	-
Follow up (d)	275 ± 265	256 ± 250	335 ± 250	0.53

* *n* = 4 patients did not reach the 30d follow up

A preoperative motor deficit was present in 7/25 (IORT) and 2/8 (adj. RT) patients respectively. Of these preexisting motor deficits, 5/24 (IORT) and 2/7 (adj. RT) patients fully recovered and 2/24 (IORT) and 0/7 (adj. RT) remained unchanged within 30 days after surgery.

**Fig. 2:**
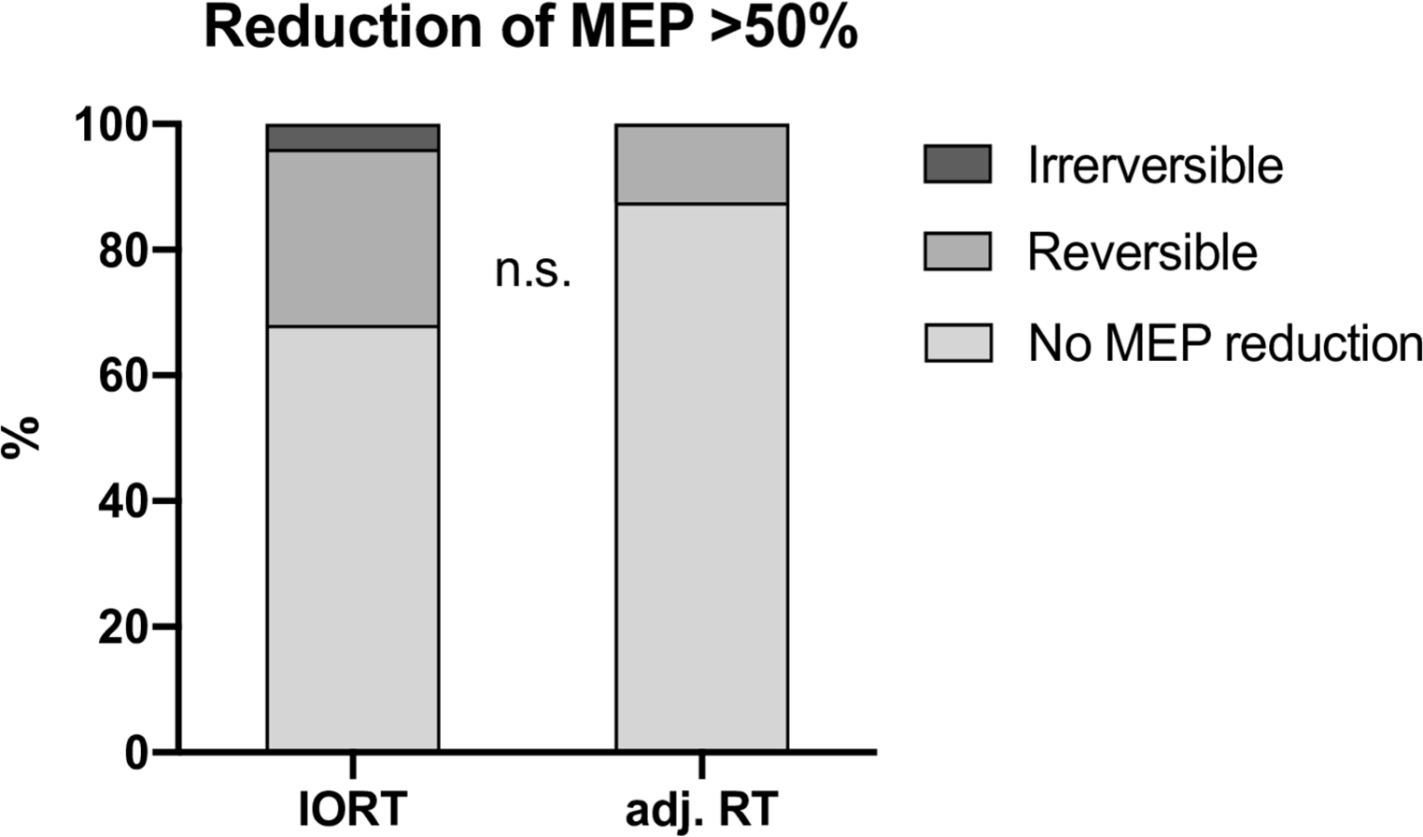
Rate of reduction of motor evoked potential (MEP) signal > 50%: dark grey = irreversible, medium grey = reversible, light grey = no MEP reduction; adj. RT = adjuvant radiotherapy, IORT = intraoperative radiotherapy; data is shown as (%), n.s. = not significant

Intraoperative neuromonitoring showed transient MEP amplitude reduction > 50% in 3/7 patients suffering from a new postoperative motor deficit (IORT: 2/4 vs. adj. RT: 1/3). The rate of MEP decline > 50% did not differ significantly between the IORT and the adj. RT group (IORT 8/25 vs. adj. RT 1/8; Chi [2]-test: *p* = 0.28) (Fig. 2; Table 3). No reduction > 50% in MEP amplitude was observed in 4/7 patients with a new postoperative motor deficit. No epileptic seizure occurred in any patient within the 30-day follow up. Persistent dysphasia was present in 5 patients (IORT *n* = 2, adh. RT *n* = 3) with 4 of them having BM on the left hemisphere. In every case, dysphasia was present before surgery and improved within the 30 day follow up only in one patient (adj. RT). Due to the unbalanced study sample we performed inverse propensity score weighting in order to correct for (age, KPS, RPA, Tumor size and duration of surgery). We did not find a significantly elevated risk of postoperative motor deficit OR 0.319 [95%CI 0.044–2.29] *p* = 0.26, or AEs OR 3.37 [95%CI 0.52–21.91] *p* = 0.204 in either group.

### Functional outcome

The functional status according to the KPS showed no significant difference between both groups before (IORT: 90% [80–90] vs. adj. RT: 85% [65–90]; Mann-Whitney-U-test: *p* = 0.29) or after surgery (IORT: 90% [72.5–90] vs. adj. RT: 80% [70–90]; Mann-Whitney-U-test: *p* = 0.31). Furthermore, no significant differences appeared, comparing KPS after radiotherapy (in IORT = after surgery) in both groups (IORT: 90% [72.5–90] vs. adj. RT: 90% [57.5–90]; Mann-Whitney-U-test: *p* = 0.64) (Table 3).

### Adverse events

**Fig. 3:**
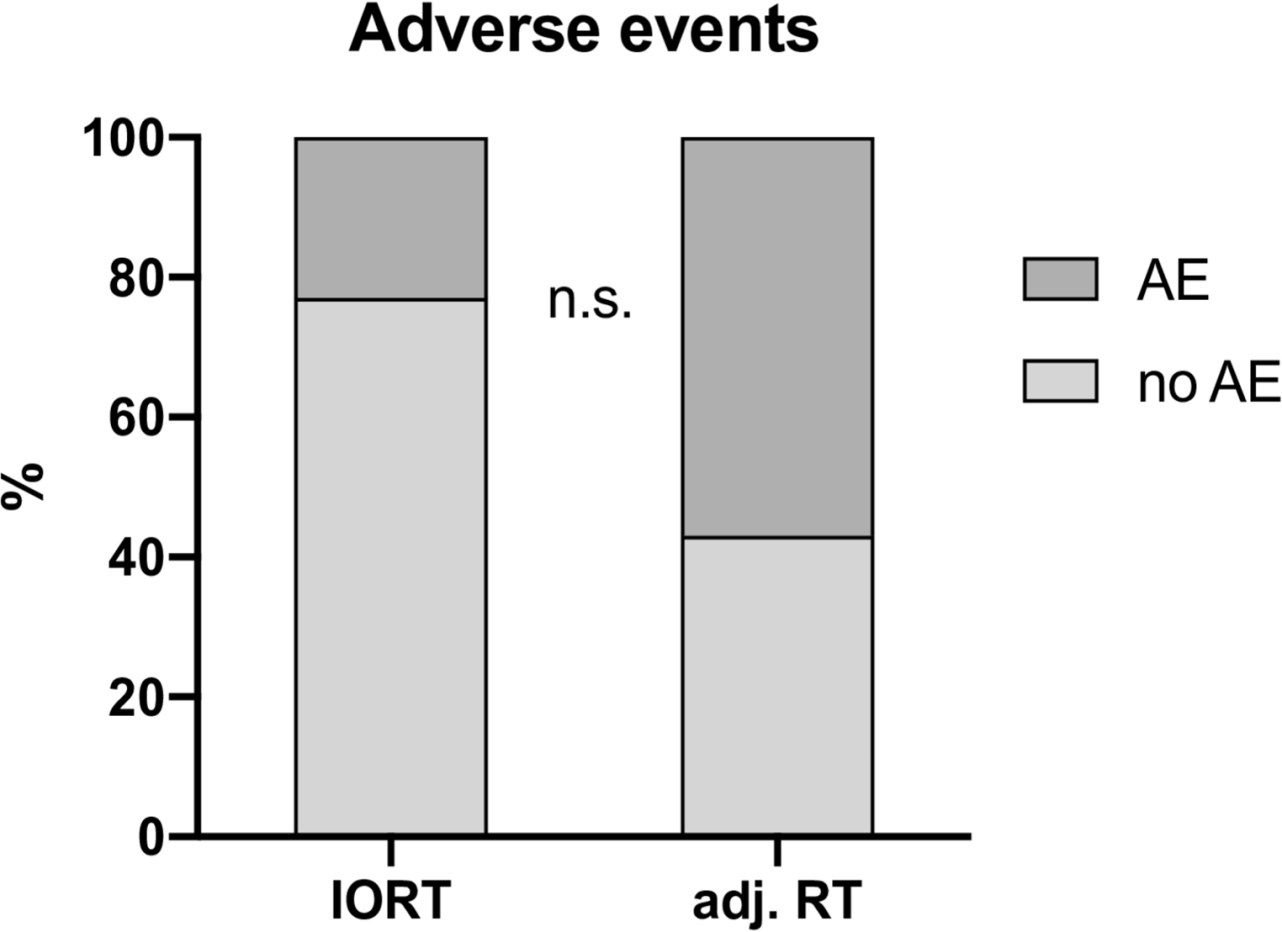
Rate of adverse events (AE): medium grey = AE, light grey = no AE; adj. RT = adjuvant radiotherapy, IORT = intraoperative radiotherapy; data is shown as (%), n.s. = not significant

No patient in both groups experienced local tumor recurrence or radio necrosis. In total 9/33 patients had postoperative adverse events (AE) within the 30 day follow up. No significantly different rate of AE was found between both groups (IORT 5/25 vs. adj. RT 4/8; Chi [2]-test: *p* = 0.09) (Fig. 3; Table 3). The Odds Ratio of having an AE (0.25 [95%CI 0.05–1.37]) favored IORT over adj. RT. Patients that experienced AEs did not show significant differences in age (*p* = 0.53), KPS before surgery (*p* = 0.50), KPS after surgery (*p* = 0.38), RPA (*p* = 0.95), Tumor size (*p* = 0.07), RT fractions (*p* = 0.09) and length of surgery (*p* = 0.74). Four deaths occurred within the 30 day follow up (IORT *n* = 3, adj. RT *n* = 1). In the IORT group, one patient died from thalamic infarction (CDG 5), another patient died from uncontrollable sepsis due to urinary tract infection (CDG 5), one patient had epidural re-bleeding after surgery and sepsis of unknown origin (CDG 5) and two patients experienced a transient new motor deficit that resolved without further surgical or pharmacological intervention (CDG 1). In the adj. RT group, one patient experienced decline of the overall status after surgery and a change of the therapeutic towards best supportive care was undertaken (CDG 5), one patient had symptomatic postoperative cerebral edema needing intravenous steroid medication (CDG 3), two patients had new transient motor deficits, that resolved without further surgical or pharmacological intervention (CDG 1). Notably, no single surgical site infection occurred in either group.

## Discussion

In this study we compared the rate of new early and late postoperative motor deficits in patients undergoing resection of motor eloquent BMs with or without IORT.

### Baseline parameters

Our study cohorts showed no significant differences in baseline parameters. The size of both cohorts did differ significantly (*n* = 25 vs. *n* = 8), which does not allow analyses that require normal distributed groups. This flaw was due to an internal shift of standard operating procedures during the time of inclusion. Only from 2020 on, IONM was established on a regular basis for BMs and gliomas. At this time, IORT has already become a local standard therapy offered to every patient undergoing surgery for BM. In this study patients that received adj. RT had either declined IORT or technical issues made IORT impossible in selected cases. Furthermore, there was a higher proportion of female patients and patients with left sided lesions that received resection with adj. RT. Whether, sex is a predictor of outcome in RT for BMs is controversially debated [14, 15, 16]. An acute impact of sex on radiosensitivity and therefore possible affection of motor tracts however has not been reported so far. In this study, the LOS did not differ significantly between both groups. This is an unintuitive finding, as brain IORT adds 10–30 min. of radiation time to a surgical procedure plus the time needed to install the IORT device. Whether this is the result of a systematic bias with BMs in the adj. RT group were surgically more demanding cannot fully be ruled out. However, size and KPS did not differ significantly between both groups.

### Functional outcome

In this study, we found no significant difference in functional outcome according to the KPS directly after surgery or 30 days after radiotherapy. The functional status is highly affecting the outcome in cancer patients [10]. The aim of any BM related intervention is to improve the functional status or at least not deteriorate it, which might hinder a comprehensive treatment. IORT has been shown to be a safe method in the treatment of BMs during the direct postoperative course [6, 7]. In the context of a comprehensive treatment, it allows faster transition to systemic therapy, as no delay between surgery and RT is needed [9]. 

### Motor eloquent lesions and motor outcome

Treatment of motor eloquent lesions is challenging [1]. Motor deficits have been shown to affect overall oncological outcome and prognosis [17, 18]. Therefore, relief of preexisting or avoidance of new motor deficits is of utmost importance if considering local treatment in this area. Application of ionizing radiation has been investigated for lesions in motor eloquent regions of the brain [19, 20, 21, 22]. Radiogenic tissue damage can be caused by deterministic and stochastic effects of ionizing radiation [23]. Radiation has been shown to increase peritumoral brain edema, which might result in malfunction of affected brain regions [24]. In this study, no difference in the rate of new motor deficits was associated to IORT. Neither paresis early after resection, nor subacute paresis with latency after surgery or radiotherapy occurred significantly more often in either group. Stereotactic Radiosurgery for motor eloquent metastases has been shown to create motor deterioration in 22–36% of cases and seem to be dose dependent [25, 26]. However, acute radiation related motor impairment is rare and was observed to resolve within a short period of time [27]. Several weeks to months after radiotherapy, neurological deficits or seizures can be associated with symptomatic radio necrosis. which results from aseptic radio induced inflammation of irradiated brain structures [28]. IORT has been shown to be associated with a low rate of radio necrosis [8, 13]. This is in line with the results of the present study in which no single patient developed a radio necrosis during follow up. The authors believe, that the location, either motor eloquent or non-motor eloquent, should not affect longer term local outcome. This includes longer term local control, which was shown to be comparable to adj. RT (90.5%) in a recent monocentric retrospective analysis by the same study group [29]. In order to better differentiate the pathophysiology of occurring motor symptoms in this study, only patients that had intraoperative neurophysiological motor monitoring (IONM) were included. IONM can predict neuronal functional decline and resection can be adapted to prevent neurological deficits [30]. No patient in either group experienced permanent decline > 50% of signal intensity, which is an indicator of irreversible functional loss. Transient decline in signal intensity can be associated with neuronal irritation but is not associated with irreversible motor deficits. In a study focusing on cortical excitation during surgery with IORT, no pathological signals could be detected [31]. Furthermore transient neurological deficits without decline in IONM can result from damage in supplementary motor areas [32]. These symptoms regularly disappear after short time [33, 34]. 

### Adverse events

Overall adverse event rate was 27%(9/33) including 7/33 experiencing new transient motor deficits. Prior reports on adverse events in BM surgery describe rates from 9 to 40%, which covers the rate found in our cohort [35, 36]. One has to keep in mind that most patients experienced transient symptoms that resolved within 30 days and only patients with motor eloquent lesions were operated. These lesions inherently have a higher rate of motor deficits and therefore AEs. Regarding the rate of fatal AEs in this cohort, the authors do not see a clear relation to the application of IORT in the respective cases. One patient died from urosepsis and one from immediate postoperative epidural hematoma. In these cases a causality in regard to IORT seems highly unlikely. One patient suffered from thalamic infarction. In this case a radiation induced vasculopathy has to be discussed, however this patient showed an intraoperative decline in MEP already before IORT and resection associated vascular damage seems more likely. Nevertheless, radiation induced changes to cerebral vascular structures have been described in the literature before but they have been assumed to appear weeks to months after radiation as result of radiation induced inflammation and alterations of endothelial cells [37]. Given the low rate of radionecrosis reported for brain IORT an elevated risk for circulation related AEs seems unlikely [29]. 

### Study limitations

This study has several limitations, that have to be clearly addressed. First, the retrospective nature of the study is inherently prone to selection bias. This is especially important in this study, as the intervention / IORT has been assigned as local standard of care. However, patients were able to deny IORT or IORT was not applied during weekend or nighttime surgery and if technical problems made this standard therapy unavailable. Second, during the study period and after implementation of IONM, IORT has been assigned as local standard procedure. Therefore a small number of patients that received adj. therapy could be used as control group. Whether radiotherapy induces transient edema around the resection cavity was not possible to investigate, as not in all patients, direct postoperative MRI scans were performed. Nevertheless, clinical meaningful edema was addressed with neurological decline as surrogate parameter. The dose of perioperative steroids did not follow a standardized protocol and was adapted according to patients symptoms and tolerance of steroid effects and side effects. The underlying oncologic disease was heterogenous in both groups. However, various entities of BMs are not known to show different postoperative courses, which is the focus of this study. Whether our findings ultimately affect the oncologic prognosis cannot be answered with this data. The lack of long-term outcome assessment including quality of life measures and the small sample size are a major limitation of this study. Studies including greater cohorts, ideally in a prospective setup and with a longer follow-up, are needed to compare both therapy strategies.

## Conclusion

In this case series we report on the effects of IORT in patients that undergo surgery for motor eloquent BMs. Our series indicates that IORT in motor eloquent regions is feasible and appears relatively safe, but larger studies with longer follow up are needed.

## Data Availability

Upon request.

## Abbreviations

adj.: RT adjuvant radiotherapy
AE: Adverse event
BM: Brain metastasis
CDG: Clavien Dindo Grading
EBRT: External beam radiotherapy
IONM: Intraoperative neuromonitoring
IORT: Intraoperative radiotherapy
KPS: Karnofsky Performance Score
LOS: Length of surgery
MEP: Motor evoked potentials
MRI: Magnet resonance imaging
RPA: Recursive partitioning analysis
RT: Radiotherapy
SD: Standard deviation
SRS: Stereotactic radiosurgery

## References

[1] Obermueller T, Schaeffner M, Shiban E et al (2015) Intraoperative neuromonitoring for function-guided resection differs for supratentorial motor eloquent gliomas and metastases. BMC Neurol 15:211 26487091 10.1186/s12883-015-0476-0 PMC4618356

[2] Le Rhun E, Guckenberger M, Smits M et al (2021) EANO-ESMO clinical practice guidelines for diagnosis, treatment and follow-up of patients with brain metastasis from solid tumours. Ann Oncol 32(11):1332–1347 34364998 10.1016/j.annonc.2021.07.016

[3] Soffietti R, Abacioglu U, Baumert B et al (2017) Diagnosis and treatment of brain metastases from solid tumors: guidelines from the European association of Neuro-Oncology (EANO). Neuro Oncol 19(2):162–174 28391295 10.1093/neuonc/now241 PMC5620494

[4] Cifarelli CP, Brehmer S, Vargo JA et al (2019) Intraoperative radiotherapy (IORT) for surgically resected brain metastases: outcome analysis of an international cooperative study. J Neurooncol 145(2):391–397 31654248 10.1007/s11060-019-03309-6 PMC7007764

[5] Giordano FA, Brehmer S, Murle B et al (2019) Intraoperative radiotherapy in newly diagnosed glioblastoma (INTRAGO): an Open-Label, Dose-Escalation phase I/II trial. Neurosurgery 84(1):41–49 29528443 10.1093/neuros/nyy018

[6] Krauss P, Kahl KH, Bonk MN et al (2023) Intraoperative radiotherapy after resection of brain metastases located in the posterior fossa. Analysis of postoperative morbidity and mortality in a single center cohort. J Clin Neurosci 118:1–6 37832264 10.1016/j.jocn.2023.09.014

[7] Krauss P, Steininger K, Motov S et al (2022) Resection of supratentorial brain metastases with intraoperative radiotherapy. Is it safe? Analysis and experiences of a single center cohort. Front Surg 9:1071804 36632525 10.3389/fsurg.2022.1071804 PMC9826792

[8] Kahl KH, Shiban E, Gutser S et al (2022) Focal cavity radiotherapy after neurosurgical resection of brain metastases: sparing neurotoxicity without compromising locoregional control. Strahlenther Onkol 198(12):1105–1111 36149437 10.1007/s00066-022-02003-3 PMC9700584

[9] Dejonckheere CS, Layer JP, Hamed M et al (2023) Intraoperative or postoperative stereotactic radiotherapy for brain metastases: time to systemic treatment onset and other patient-relevant outcomes. J Neurooncol 164(3):683–691 37812290 10.1007/s11060-023-04464-7 PMC10589145

[10] Schag CC, Heinrich RL, Ganz PA (1984) Karnofsky performance status revisited: reliability, validity, and guidelines. J Clin Oncol 2(3):187–193 6699671 10.1200/JCO.1984.2.3.187

[11] Dindo D, Demartines N, Clavien PA (2004) Classification of surgical complications: a new proposal with evaluation in a cohort of 6336 patients and results of a survey. Ann Surg 240(2):205–213 15273542 10.1097/01.sla.0000133083.54934.ae PMC1360123

[12] Mak PH, Campbell RC, Irwin MG (2002) American society of A. The ASA physical status classification: inter-observer consistency. American society of anesthesiologists. Anaesth Intensive Care 30(5):633–640 12413266 10.1177/0310057X0203000516

[13] Kahl KH, Balagiannis N, Hock M et al (2021) Intraoperative radiotherapy with low-energy x-rays after neurosurgical resection of brain metastases-an Augsburg university medical center experience. Strahlenther Onkol 197(12):1124–1130 34415358 10.1007/s00066-021-01831-z PMC8604815

[14] Jung KW, Park S, Shin A et al (2012) Do female cancer patients display better survival rates compared with males? Analysis of the Korean National registry data, 2005–2009. PLoS ONE 7(12):e52457 23300677 10.1371/journal.pone.0052457 PMC3530449

[15] Mangesius J, Seppi T, Bates K et al (2021) Hypofractionated and single-fraction radiosurgery for brain metastases with sex as a key predictor of overall survival. Sci Rep 11(1):8639 33883632 10.1038/s41598-021-88070-5 PMC8060341

[16] Cioffi G, Ascha MS, Waite KA et al (2024) Sex differences in odds of brain metastasis and outcomes by brain metastasis status after advanced melanoma diagnosis. Cancers (Basel). 16(9) 10.3390/cancers16091771 PMC11083203 38730723

[17] Tang V, Rathbone M, Park Dorsay J, Jiang S, Harvey D (2008) Rehabilitation in primary and metastatic brain tumours: impact of functional outcomes on survival. J Neurol 255(6):820–827 18500499 10.1007/s00415-008-0695-z

[18] McGirt MJ, Mukherjee D, Chaichana KL, Than KD, Weingart JD, Quinones-Hinojosa A (2009) Association of surgically acquired motor and Language deficits on overall survival after resection of glioblastoma multiforme. Neurosurgery 65(3):463–469 discussion 469–470 19687690 10.1227/01.NEU.0000349763.42238.E9

[19] Ding D, Yen CP, Xu Z, Starke RM, Sheehan JP (2013) Radiosurgery for primary motor and sensory cortex arteriovenous malformations: outcomes and the effect of eloquent location. Neurosurgery 73(5):816–824 discussio 824 23867301 10.1227/NEU.0000000000000106

[20] Diehl CD, Rosenkranz E, Schwendner M et al (2022) Dose reduction to motor structures in adjuvant fractionated stereotactic radiotherapy of brain metastases: nTMS-Derived DTI-Based motor fiber tracking in treatment planning. Cancers (Basel). 15(1) 10.3390/cancers15010282 PMC9818359 36612277

[21] Ruge MI, Kickingereder P, Grau S et al (2013) Stereotactic iodine-125 brachytherapy for the treatment of WHO grades II and III gliomas located in the central sulcus region. Neuro Oncol 15(12):1721–1731 24046261 10.1093/neuonc/not126 PMC3829594

[22] Dzierma Y, Schuermann M, Melchior P et al (2021) Optimizing adjuvant stereotactic radiotherapy of motor-Eloquent brain metastases: sparing the nTMS-Defined motor cortex and the hippocampus. Front Oncol 11:628007 33718201 10.3389/fonc.2021.628007 PMC7953904

[23] Turnquist C, Harris BT, Harris CC (2020) Radiation-induced brain injury: current concepts and therapeutic strategies targeting neuroinflammation. Neurooncol Adv 2(1):vdaa057 32642709 10.1093/noajnl/vdaa057 PMC7271559

[24] Harat M, Lebioda A, Lasota J, Makarewicz R (2017) Evaluation of brain edema formation defined by MRI after LINAC-based stereotactic radiosurgery. Radiol Oncol 51(2):137–141 28740448 10.1515/raon-2017-0018 PMC5514653

[25] Luther N, Kondziolka D, Kano H, Mousavi SH, Flickinger JC, Lunsford LD (2013) Motor function after stereotactic radiosurgery for brain metastases in the region of the motor cortex. J Neurosurg 119(3):683–688 23870018 10.3171/2013.6.JNS122081

[26] Park CY, Choi HY, Lee SR, Roh TH, Seo MR, Kim SH (2016) Neurological change after gamma knife radiosurgery for brain metastases involving the motor cortex. Brain Tumor Res Treat 4(2):111–115 27867921 10.14791/btrt.2016.4.2.111 PMC5114181

[27] Tofilon PJ, Fike JR (2000) The radioresponse of the central nervous system: a dynamic process. Radiat Res 153(4):357–370 10798963 10.1667/0033-7587(2000)153[0357:trotcn]2.0.co;2

[28] Rahmathulla G, Marko NF, Weil RJ (2013) Cerebral radiation necrosis: a review of the pathobiology, diagnosis and management considerations. J Clin Neurosci 20(4):485–502 23416129 10.1016/j.jocn.2012.09.011

[29] Kahl KH, Krauss PE, Neu M et al (2024) Intraoperative radiotherapy after neurosurgical resection of brain metastases as institutional standard treatment- update of the oncological outcome form a single center cohort after 117 procedures. J Neurooncol 169(1):187–193 38963657 10.1007/s11060-024-04691-6 PMC11269407

[30] Rossi M, Sciortino T, Conti Nibali M et al (2021) Clinical pearls and methods for intraoperative motor mapping. Neurosurgery 88(3):457–467 33476393 10.1093/neuros/nyaa359 PMC7884143

[31] Cifarelli CP, Vargo JA, Sener U, Cifarelli DT, Scoville D, Dabir A (2023) Intracranial intraoperative radiotherapy (IORT): evaluation of electrocorticography and peri-operative seizure risk. J Neurooncol 164(2):423–430 37668944 10.1007/s11060-023-04443-y

[32] Giampiccolo D, Parisi C, Meneghelli P et al (2021) Long-term motor deficit in brain tumour surgery with preserved intra-operative motor-evoked potentials. Brain Commun 3(1):fcaa226 33615216 10.1093/braincomms/fcaa226 PMC7884605

[33] Palmisciano P, Haider AS, Balasubramanian K et al (2022) Supplementary motor area syndrome after brain tumor surgery: A systematic review. World Neurosurg 165:160–171e162 35752423 10.1016/j.wneu.2022.06.080

[34] Nakajima R, Kinoshita M, Yahata T, Nakada M (2019) Recovery time from supplementary motor area syndrome: relationship to postoperative day 7 paralysis and damage of the cingulum. J Neurosurg 132(3):865–874 30738403 10.3171/2018.10.JNS182391

[35] Jakola AS, Gulati S, Nerland US, Solheim O (2011) Surgical resection of brain metastases: the prognostic value of the graded prognostic assessment score. J Neurooncol 105(3):573–581 21660540 10.1007/s11060-011-0623-4 PMC3215882

[36] Wong JM, Panchmatia JR, Ziewacz JE et al (2012) Patterns in neurosurgical adverse events: intracranial neoplasm surgery. Neurosurg Focus 33(5):E16 23116096 10.3171/2012.7.FOCUS12183

[37] O’Connor MM, Mayberg MR (2000) Effects of radiation on cerebral vasculature: a review. Neurosurgery 46(1):138–149 discussion 150– 131 10626944 10.1093/neurosurgery/46.1.138

